# Neural Entrainment vs. Stimulus-Tracking: A Conceptual Challenge for Rhythmic Perceptual Stimulation in Developmental Neuroscience

**DOI:** 10.3389/fpsyg.2022.878984

**Published:** 2022-05-06

**Authors:** Anna Bánki, Alicja Brzozowska, Stefanie Hoehl, Moritz Köster

**Affiliations:** ^1^Faculty of Psychology, University of Vienna, Vienna, Austria; ^2^Institute of Psychology, University of Regensburg, Regensburg, Germany; ^3^Faculty of Education and Psychology, Freie Universität Berlin, Berlin, Germany

**Keywords:** neural entrainment, stimulus-tracking, rhythmic perceptual stimulation, steady-state response (SSR), frequency-tagging, cognitive development

## Introduction

Rhythmic perceptual stimulation, i.e., the presentation of periodic stimuli via sensory input pathways (e.g., auditory or visual; Thut et al., 2011; Calderone et al., 2014) induces resonant brain responses at the presentation frequencies. Since rhythmic perceptual stimulation does not perturbate the neural system beyond its normal operating range (Obleser and Kayser, 2019) and is non-invasive, it has also become a promising tool for neuromodulation in cognitive and developmental neuroscience. In particular, recent work focused on applying rhythmic perceptual stimulation to uncover the functional relevance of neural oscillations at specific frequencies, and their links with cognitive processes in early development, including attention (Christodoulou et al., 2018) and learning (Köster et al., 2019a).

However, an open debate in the adult literature (e.g., Keitel et al., 2014; Haegens, 2020; Meyer et al., 2020; Doelling and Assaneo, 2021; van Bree et al., 2022) emphasizes the need to ascertain whether rhythmic perceptual stimulation directly alters (i.e., stimulates) intrinsic brain oscillations, a phenomenon referred to as *entrainment*, or rather elicits a series of perceptually evoked potentials independent of endogenous oscillatory activity, referred to as *stimulus-tracking* (Capilla et al., 2011; Notbohm et al., 2016).

There is a growing body of research with adults showing that perceptual entrainment of endogenous neural oscillations is indeed possible (Herrmann, 2001; Haegens and Zion Golumbic, 2018; Lakatos et al., 2019; Obleser and Kayser, 2019), as demonstrated by its effects on behavioral outcomes such as memory performance (Köster et al., 2019b) and temporal predictions (Daume et al., 2021), and its interplay with individual intrinsic frequencies (Notbohm et al., 2016; Gulbinaite et al., 2019). Yet, conclusive evidence for entrainment is still lacking, especially from developmental populations.

Here, we bring forward the challenges of interpreting results from rhythmic perceptual stimulation studies with infants and children and discuss how insights from the adult literature can help us adequately examine the entrainment hypothesis in developmental research. We argue that neural entrainment is indeed possible in the developing brain, but that further critical evidence is needed to pin down the underlying neural mechanisms. We discuss the implications of distinguishing entrainment from stimulus tracking for the application of rhythmic perceptual stimulation in developmental neuroscience and suggest potential avenues for future research.

## Entrainment vs. Stimulus-Tracking

Oscillations can be modified through synchronization with an external periodic stimulus, a phenomenon called entrainment. By definition, during entrainment, endogenous neural oscillations align with the temporal structure of the exogenous stimulus (Thut et al., 2011; Obleser and Kayser, 2019). It has been proposed that entrainment may facilitate information sampling and sensory selection, underlying various cognitive and perceptual processes (Lakatos, 2008).

**Figure 1:**
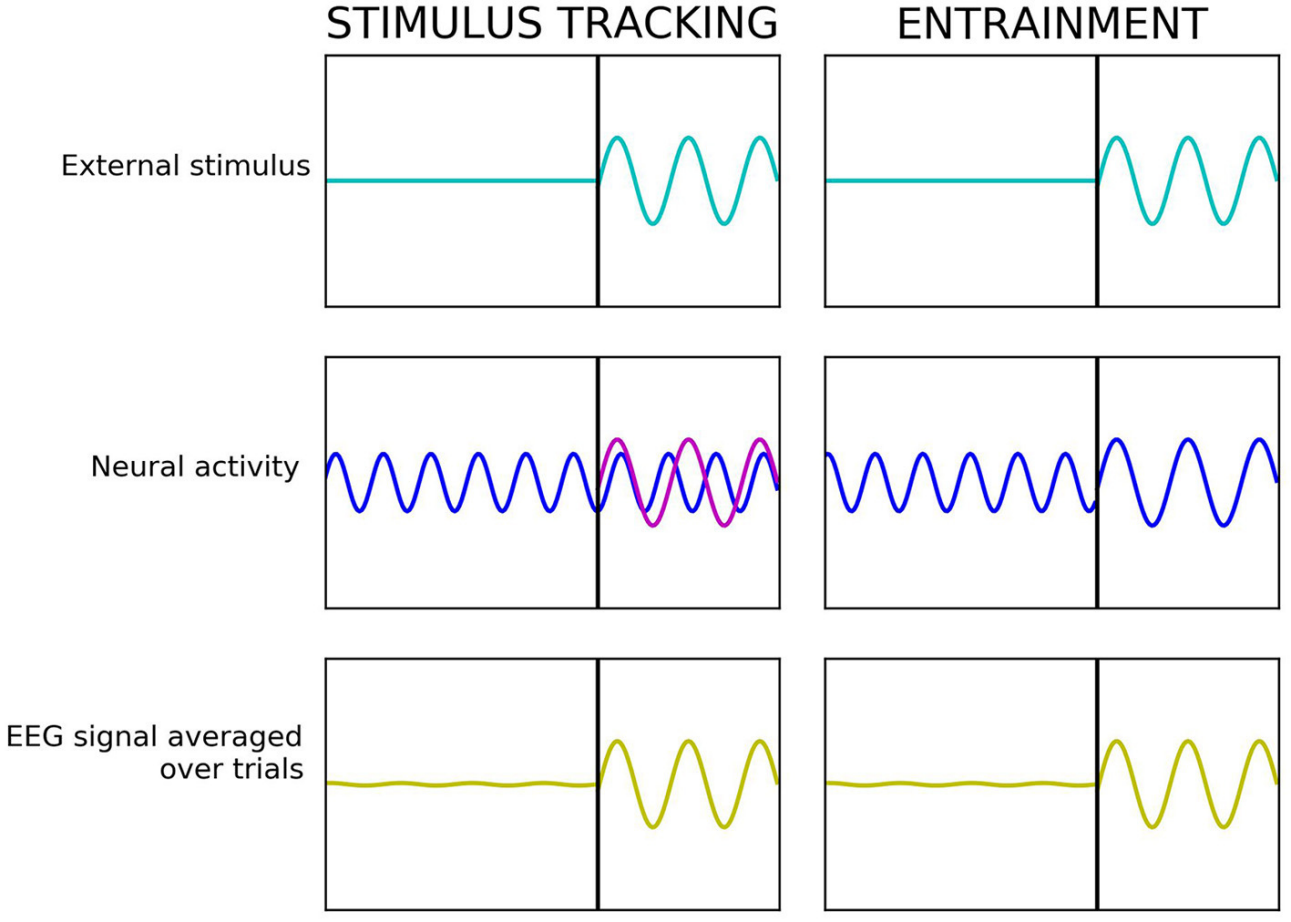
Stimulus-tracking vs. entrainment—simplified example. In all the panels, the *x*-axis reflects the time and the *y*-axis—the amplitude of the signal. In stimulus-tracking (left), rhythmic external stimulation (light blue) causes a frequency-following neural sensory response (magenta), but the internal rhythms (dark blue) are not perturbated. In entrainment (right), rhythmic external stimulation (light blue) causes a resonant response at the frequency of the external stimulus by “hijacking” ongoing internal rhythms (dark blue). In both cases, the resulting electroencephalogram (EEG) signal (yellow) looks the same.

Stimulus-tracking refers to the occurrence of frequency-following brain responses to a rhythmic stimulus that, unlike entrainment, show no direct interference with ongoing internal oscillations and related cognitive and perceptual processes (Keitel et al., 2014, 2019; Haegens, 2020). The main difference between the two phenomena is that during stimulus-tracking, a rhythmic external stimulus elicits a frequency following neural sensory response, but ongoing internal brain rhythms are not perturbated. Whereas during entrainment, the stimulus “hijacks” the ongoing internal rhythms at the stimulation frequency, which then become altered and align with the external stimulus (Figure 1).

The phenomenon of stimulus-tracking forms the basis of frequency-tagging, a method commonly applied in visual perception research: tagging multiple, simultaneously presented stimuli with different frequencies elicits steady-state responses (SSRs), thus the visual processing of each element can be assessed separately (Müller et al., 2003). Importantly, frequency-tagging considers SSRs independent from the preferred oscillatory frequency of the involved sensory networks (Keitel et al., 2014).

Analytically distinguishing between entrainment and stimulus-tracking solely based on brain activity during rhythmic perceptual stimulation is not possible, as both are characterized by the synchronization between an exogenous stimulus and neural rhythms (Thut et al., 2011; Obleser and Kayser, 2019; Haegens, 2020). However, differentiating between the two phenomena is crucial for studies aiming to cause neuromodulation effects or to test hypotheses about the functionality of endogenous brain rhythms through applying the method of rhythmic perceptual stimulation. There are also implications for paradigms that rely on this methodology to assess attention and perception processes without a specific focus on the underlying oscillatory activity.

Some have postulated that for entrainment to be differentiated from phase-locked responses, it would require that endogenous neural oscillations decouple from the external stimulus and outlive it, particularly in the narrow frequency band in which these oscillations operate (Capilla et al., 2011; Haegens and Zion Golumbic, 2018), but it is not a universally accepted criterion (see e.g., Doelling and Assaneo, 2021). Nonetheless, alterations of endogenous oscillations would likely manifest on the behavioral level and thus could be experimentally tested (Lakatos, 2008).

In the adult literature, there have been two significant ways in particular to establish entrainment: by the alteration of behavioral performance, and by the alignment/matching between the stimulation frequency and the individual internal oscillations of participants. In the following, we discuss both methods and outline associated challenges as well as future directions for entrainment research with infants and children.

## Behavioral Evidence for Entrainment

Endogenous brain rhythms at different frequency bands have differing functional correlates, e.g., the theta band has been linked to memory processes (Klimesch, 1999; Friese et al., 2013), while the alpha band has been associated with attentional processes (Klimesch, 2012). If rhythmic perceptual stimulation is able to entrain these endogenous brain rhythms, then we should be able to observe distinct behavioral effects of stimulation at external frequencies corresponding to different EEG frequency bands (Herrmann et al., 2016).

Good examples of this line of research supporting this notion are studies examining memory-enhancing effects of rhythmic sensory stimulation at the theta frequency, which have accumulated in the recent years (see Hanslmayr et al., 2019 for a review). For example, it has been shown that presenting audiovisual and visual stimuli at the theta, but not the alpha frequency improves later memory of these stimuli (Clouter et al., 2017; Köster et al., 2019b). What is important, this effect is dependent on the phase synchrony between the external stimulus and the brain response (Clouter et al., 2017; Wang et al., 2018), as well as the power of the response (Köster et al., 2019b), which further suggests that the behavioral effects of rhythmic perceptual stimulation are caused by neural entrainment. Interestingly, the behavioral effects of entrainment of the theta rhythm seem to persist beyond the period of stimulation, as Roberts et al. (2018) found improved source memory in participants exposed to rhythmic sensory stimulation (content-wise irrelevant to the memorized stimuli) between learning and test phase. To conclude, research with adults provides accumulating evidence for the modulation of cognitive processes through rhythmic perceptual stimulation.

## Entrainment at Individual Stimulation Frequencies

It has been argued that an important feature of entrainment is that it should occur for external rhythms close to the neural oscillator's intrinsic rate (so-called “eigenfrequency”; Obleser and Kayser, 2019; Haegens, 2020). Indeed, Notbohm et al. (2016) demonstrated that rhythmic visual flicker at frequencies closer to participants' intrinsic frequencies of the alpha rhythm caused a more pronounced phase coupling between the external rhythm and the neural response. Moreover, a number of studies have found that the behavioral effects (i.e., performance impairment) of stimulation in the alpha band on participants' attention are predicted by how close the stimulation frequencies are to participants' endogenous alpha peaks (de Graaf et al., 2013; Gulbinaite et al., 2017). Finally, Köster et al. (2019b) successfully entrained memory functions by stimulating participants' brain activity at their individual theta vs. alpha frequencies.

However, more studies examining other frequency bands and an improved understanding of the generators behind the intrinsic brain rhythms are needed. Ideally, future studies would also examine the behavioral effects of individually optimized vs. not individually optimized stimulation frequencies within the same frequency bands. In developmental populations, EEG frequency ranges change with age by moving toward higher frequencies (Marshall et al., 2002), thus we would also expect individual frequencies to shift toward the higher ends of the spectra with age. Observing behavioral effects of rhythmic sensory stimulation at frequencies optimized (vs. non-optimized) to specific age groups would support the notion of entrainment effects.

## Entrainment in Developmental Research

Previous studies with infants and children applied rhythmic auditory and/or visual stimulation as a tool to assess attention (e.g., Robertson et al., 2012; Köster et al., 2017; Christodoulou et al., 2018), face perception (e.g., de Heering and Rossion, 2015; Peykarjou et al., 2017), speech perception and language learning (e.g., Telkemeyer et al., 2009; Attaheri et al., 2022), and related developmental disorders such as dyslexia (e.g., Colling et al., 2017), as well as musical rhythm perception (e.g., Cirelli et al., 2016). In these studies, stimulation frequencies were chosen either arbitrarily, to index brain responses to stimuli of interest (e.g., a face appearing at 1.2 Hz among a stream of images updated at 6 Hz in the study by de Heering and Rossion, 2015), or were motivated by hypotheses about naturally occurring frequencies in infants' environment (e.g., sounds with spectral properties resembling naturalistic speech in the study by Telkemeyer et al., 2009). Another approach aimed to capture infants' stimulus-tracking by reconstructing features of natural stimuli based on brain responses or by predicting brain activity from stimulus features (e.g., Jessen et al., 2021). However, very few developmental studies to date applied rhythmic visual stimulation with the explicit goal to entrain endogenous rhythms (e.g., Köster et al., 2019a), an approach that can help to uncover the functionality of brain oscillations in early cognitive and perception processes.

Recent debates in the developmental literature challenged the interpretation of findings from rhythmic perceptual stimulation studies since the underlying mechanisms of entrainment are still not well-understood. As an example, Köster et al. (2019a) used this method to assess the functional role of infants' theta (4 Hz) and alpha (6 Hz) oscillations in the cognitive processing of unexpected events. Findings revealed that infants' visually entrained theta, but not alpha oscillations sharply increased for unexpected vs. expected events, in line with evidence on the critical role of theta oscillations in early learning (Begus and Bonawitz, 2020; Köster et al., 2020). However, in a commentary, using simulated data, Keitel et al. (2021) argued that the results from Köster et al. (2019a) could potentially be explained by a stimulus-tracking account: unexpected events could enhance the negative central (Nc) component of the event related potential (ERP) (Kayhan et al., 2019), leading to a difference in ERPs between conditions without the involvement of functionally relevant oscillations elicited by the stimulus. Although the simulated data did not closely reflect the observed data (see Köster et al., 2021b), this debate highlights the need to differentiate between entrainment and stimulus-tracking when interpreting data from rhythmic perceptual stimulation studies.

## Avenues for Future Research

Here, we argued that further evidence is needed on how perceptually entrained rhythms interact with ongoing oscillatory dynamics in the developing brain, so that a comprehensive theoretical and analytical framework could be established for developmental entrainment research. Studies in the field need to investigate the effects of rhythmic perceptual stimulation by including behavioral outcome measures, and by using individually optimized stimulation frequencies. However, commonly applied paradigms in adult entrainment research need to be adjusted for developmental studies (Wass et al., 2022).

Measuring altered behavior of infants following rhythmic perceptual stimulation—though challenging—can be achieved by subsequent memory paradigms that assess learning and memory performance via preferential looking (Begus et al., 2015), imitation (Köster et al., 2021a), or habituation and dishabituation (Choi et al., 2020). In case of older children, behavioral outcomes of entrainment such as attention, learning and memory effects can be tested by incorporating subsequent playful tasks into the experimental design. Observed behavioral effects linked to sensory stimulation would provide causal evidence for neural oscillatory entrainment. Applying stimulation at individualized frequencies could be another non-invasive way to ascertain entrainment in developmental samples, by testing the alignment of endogenous oscillations following sensory stimulation with infants' or children's individual vs. other frequencies. The two approaches can be combined to contrast behavioral changes following entrainment to individualized vs. other frequencies.

Taken together, we need a deeper understanding of the effects of rhythmic perceptual stimulation on endogenous oscillatory activity to establish if entrainment occurs in the developing brain, and if so, whether it can be considered as a tool for neuromodulation to test the functional aspects of specific neural rhythms in early cognitive development.

## Author Contributions

ABá and ABr wrote the manuscript. SH and MK contributed to revising and editing the manuscript. All authors contributed to the article and approved the submitted version.

## Funding

This work was supported by an FWF grant awarded to SH (Grant number: P 33853) and a D-A-CH research grant awarded to MK and SH by the DFG and FWF jointly (Grant numbers: KO 6028/1-1; I 4332). We are grateful for the open access funding provided by the University of Vienna.

## Conflict of Interest

The authors declare that the research was conducted in the absence of any commercial or financial relationships that could be construed as a potential conflict of interest.

## Publisher's Note

All claims expressed in this article are solely those of the authors and do not necessarily represent those of their affiliated organizations, or those of the publisher, the editors and the reviewers. Any product that may be evaluated in this article, or claim that may be made by its manufacturer, is not guaranteed or endorsed by the publisher.
